# A human iPSC-derived midbrain neural stem cell model of prenatal opioid exposure and withdrawal: A proof of concept study

**DOI:** 10.1371/journal.pone.0319418

**Published:** 2025-04-01

**Authors:** Rhea Sullivan, Quinn Ahrens, Sara L. Mills-Huffnagle, Irina A. Elcheva, Steven D. Hicks

**Affiliations:** 1 Department of Pediatrics, Penn State College of Medicine, Hershey, Pennsylvania, United States of America; 2 Department of Neural and Behavioral Sciences, Penn State College of Medicine, Hershey, Pennsylvania, United States of America; Nathan S Kline Institute, UNITED STATES OF AMERICA

## Abstract

A growing body of clinical literature has described neurodevelopmental delays in infants with chronic prenatal opioid exposure and withdrawal. Despite this, the mechanism of how opioids impact the developing brain remains unknown. Here, we developed an *in vitro* model of prenatal morphine exposure and withdrawal using healthy human induced pluripotent stem cell (iPSC)-derived midbrain neural progenitors in monolayer. To optimize our model, we identified that a longer neural induction and regional patterning period increases expression of canonical opioid receptors mu and kappa in midbrain neural progenitors compared to a shorter protocol (*OPRM1*, two-tailed t-test, *p* =  0.004; *OPRK1*, *p* =  0.0003). Next, we showed that the midbrain neural progenitors derived from a longer iPSC neural induction also have scant toll-like receptor 4 (TLR4) expression, a key player in neonatal opioid withdrawal syndrome pathophysiology. During morphine withdrawal, differentiating neural progenitors experience cyclic adenosine monophosphate overshoot compared to cell exposed to vehicle (*p* =  0.0496) and morphine exposure conditions (*p*, =  0.0136, 1-way ANOVA). Finally, we showed that morphine exposure and withdrawal alters proportions of differentiated progenitor cell fates (2-way ANOVA, F =  16.05, p <  0.0001). Chronic morphine exposure increased proportions of nestin positive progenitors (*p* =  0.0094), and decreased proportions of neuronal nuclear antigen positive neurons (NEUN) (*p* =  0.0047) compared to those exposed to vehicle. Morphine withdrawal decreased proportions of glial fibrillary acidic protein positive cells of astrocytic lineage (*p* =  0.044), and increased proportions of NEUN-positive neurons (*p* <  0.0001) compared to those exposed to morphine only. Applications of this paradigm include mechanistic studies underscoring neural progenitor cell fate commitments in early neurodevelopment during morphine exposure and withdrawal.

## Introduction

There is a growing body of literature characterizing short and long-term neurodevelopmental effects associated with prenatal opioid exposure (POE) in infants [1–3]. Short-term observations include smaller head circumferences at birth [4] (with 30% of newborns below the 10^th^ percentile [5]), visual and oculomotor issues [6–8], a heightened sympathetic arousal [9,10], and seizures [11]. Long-term effects seen in childhood and adolescence include speech, auditory, and language delays [12–15], motor and cognitive deficits [12,13,15–17], behavioral and emotional dysregulation [16], visual abnormalities [18,19], deteriorating school performance [20], decreased brain regional volumes [19,21], decreased fractional anisotropy on white matter tracts [22], and seizures [23]. Further evidence demonstrating the delayed emergence of developmental issues from POE can be found in longitudinal studies of healthcare utilization. Children with POE display significantly higher healthcare costs during the onset of elementary education (years 5, 7, and 8) compared to matched controls [24,25].

Animal models of POE demonstrate some phenotypic similarities to affected infants [26,27]. These models have been valuable proxies for investigating the effect of POE on neurodevelopment and behavior [28]. Additionally, animal models offer the ability to study POE in molecular [26], brain connectivity [29], and whole organ pathophysiology [30,31]. While the field of POE has been greatly advanced by animal models, their limitations should also be considered [32,33]. Critical caveats include: an overall protracted time of development (40 weeks gestation in humans versus 3 weeks in rodents), a lack of neural progenitor heterogeneity [34], low expression of canonical opioid receptors on mouse neural stem cells [35], missing neuronal cell types [36,37], and primitive astrocytic size and function [38–40]. It has been proposed that the brain of a newborn rodent is at the same developmental stage of a late 2^nd^ trimester fetus [41,42]. Complicating matters further, there are inconsistent recommendations on when the neonatal rodent is comparable to a human newborn, ranging from postnatal day 7 (PN7) to PN12 [27,43]. Because parallel developmental stage comparisons are difficult to make, designing translatable opioid exposure and withdrawal windows for animal experiments can be challenging [26,44]. Further, key transcriptional regulators of cell fate in the developing brain have been shown to distinguish human neurodevelopmental biology from other primates [45,46].

For these reasons, it may be useful to complement preclinical animal models of POE with human stem cell models [28,32,34,47,48]. Modeling human neurodevelopment using human induced pluripotent stem cell (hiPSC)-derived cells is an ethical, non-invasive and highly translatable tool, since neural differentiation protocols mimic endogenous embryonic signaling and regional brain patterning [49,50]. These systems have advanced our understanding of diseases such as schizophrenia, Rett syndrome, Fragile X syndrome, and fetal alcohol syndrome [49,51]. Despite calls for multifaceted approaches in POE research [32,52,53], human stem cell models are largely absent in this field [54,55]. This may be due, in part, to earlier, popular human neural progenitor cell lines losing key protein expression, like opioid receptors mu (OPRM1), kappa (OPRK1), and delta (OPRD1) and display of aberrant growth likely due to immortalization [56,57]. This concern is abrogated by the use of hiPSC-derived neural cells [58], which can be achieved with two-dimensional (2D) or three-dimensional (3D) models.

Strengths of 3D designs include organoid self-organization and the generation of diverse cell types, which more closely recapitulate developing brain tissue structure [59]. 3D studies have been useful for generating large-scale “-omics” results and electrophysiological characterizations [60–63]. Challenges of 3D culture protocols include low organoid-to-organoid reproducibility [49]. While monolayer cultures (2D) have been referred to as “foundational” or described as offering “technical contributions” [53], they are preferable for mechanistic studies due to uniformity of cell types and ease of genomic manipulation [49]. Additionally, monolayer cultures are highly reproducible, scalable, can be optimized for co-culture of two or more cell types, and are relatively inexpensive compared to 3D systems [49]. Further, CRISPR modification of patient-derived iPSC lines for generation of isogenic controls or mutation correction is more feasible in 2D compared to 3D, which is highly relevant to personalized medicine applications [64].

To our knowledge, there are eight studies examining the effects of opioids on hiPSC-derived neural development (i.e., lines not derived from adult opioid use disorder patients), all of which have been conducted in 3D culture [53]. The midbrain remains an underrepresented brain region in hiPSC-derived opioid exposure models [53], which is surprising given its role in reward, goal-directed behaviors, and its dopaminergic projections to the amygdala (emotion processing), medial prefrontal cortex (attention, inhibitory control, memory), and striatum (motor control) [65,66]. Additional evidence suggests that the midbrain dysfunction may underlie pathophysiology of psychiatric disorders [66,67]. While morphine treatments in opioid exposure models are less popular, chronic prenatal morphine and hydromorphone exposure remains a common cause of severe NOWS [68], and potentially increase risk of later neurodevelopmental delays [69,70]. Only one study using hiPSC-derived neural stem cells included the effects of opioid *withdrawal* in addition to exposure [71]. Including a withdrawal treatment group (i.e., *in vitro* morphine washout after a chronic exposure) is critical because the differing molecular mechanisms of chronic opioid agonism and opioid withdrawal have been well-defined [72,73]. Despite one study including a withdrawal treatment group, astrocytes were not generated within their reported organoids [71]. Studying the role of astrocytes in POE is critical; astrocytes provide metabolic and trophic support to developing neurons [74,75] and mediate neuroinflammation [76]. Given the recent studies of mitochondrial dysfunction in opioid withdrawal [77] and dysregulated inflammatory signaling in infants with POE [78], studying co-developing human neurons and astrocytes in culture is critical.

In this study, we developed an *in vitro* model of prenatal opioid exposure and withdrawal in a reproducible monolayer that supports co-culture of developing iPSC-derived human cells of neuronal and astrocytic lineage. This design models prenatal opioid exposure during multiple stages of neurodevelopment. These stages include regional neural progenitor patterning, cell fate commitments (i.e., during differentiation), and neuronal maturation. Further, we include a withdrawal treatment condition to model how cell fate decisions are altered in *in utero* withdrawal after chronic exposure, compared to continual morphine treatment. It is our hope that this model may serve as a foundation for future mechanistic studies interrogating cell fate alterations during POE.

## Materials and methods

### Human induced pluripotent stem cell line and culture

STEMCELL Technologies Human Control Female iPSC Line SCTi003-A was purchased from the manufacturer. SCTi003-A was derived from peripheral blood mononuclear cells from a 48 year old White female, with calculated 78.2% European and 21.8% South Asian ancestry. Human iPSCs were cultured on 0.16 mg/mL Geltrex™ LDEV-Free Reduced Growth Factor Basement Membrane Matrix (Gibco, NY) in sterile mTesR™ Plus media (STEMCELL Technologies, Vancouver) in a humidified incubator (5% CO_2_, 37°C). iPSCs were passaged as aggregates with ReLesR™ (STEMCELL Technologies, Vancouver) at 70% confluency. mFreSR™ (STEMCELL Technologies, Vancouver) was used for cryopreservation. SCTi003-A has been extensively characterized for trilineage differentiation capacity, copy number variations, and normal karyotyping by the manufacturer [79,80]. Only iPSCs with under 10 passages were used for differentiation experiments.

### Neural induction, midbrain patterning and differentiation

To determine the optimal length for neural induction and midbrain patterning for our model of POE and withdrawal (treatment groups defined below under ‘Morphine treatments and defining morphine withdrawal’), we compared opioid receptor expression at the midbrain neural progenitor stage using shorter vs. longer neural induction and regional patterning methods [81,82]. As an example of a shorter method, we followed a novel protocol optimizing the time and generation of iPSC-derived midbrain patterned neurons, herein referred to as the “shorter” method [82]. This method completed iPSC neural induction and midbrain regional patterning of neural progenitors by 13 days *in vitro*. Briefly, iPSCs were dissociated to single cells using Accutase™ and plated on Laminin-521 (5 μg/mL) in DMEM/F-12 and Neurobasal media (1:1), supplemented with Y-27632 (10 μM), SB431542 (10 μM), LDN193189 (200 nM), B27 -vitamin A (1x), N2 (1x), GlutaMAX (1x), and penicillin/streptomycin (0.5x). Y-27632 was removed from the media for daily feeding. Ventralizing factors sonic hedgehog (100 ng/mL) and purmorphamine (2 μM), plus caudalizing factor CHIR99021 (2.5 μM) were added to the neural induction media between days 2 through 10 after plating, as described [82]. On day 11 through 40, media was changed to DMEM/F-12 and Neurobasal media (1:1), B27 + vitamin A (1x), N2 (1x), non-essential amino acids (1x), insulin-transferrin-selenium-sodium pyruvate (ITS-A) (1x), GlutaMAX (1x), penicillin/streptomycin (0.5x) and supplemented with recombinant human brain-derived neurotrophic factor (rhBDNF) (20 ng/mL), recombinant human glial cell line-derived neurotrophic factor (rhGDNF) (20 ng/mL), dibutyryl cAMP (0.1 mM), ascorbic acid (200 nM), recombinant human transforming growth factor β3 (TGFβ3) (1 ng/mL), DAPT (10 μM) and CHIR99021 (2.5 μM).

As an example of a protocol utilizing a longer neural induction and patterning process, we also followed a method with a 3-week iPSC neural induction process, followed by a 2 week midbrain patterning step [81]. This protocol will herein be referred to as the “longer” neural induction method, as these progenitors completed neural induction and regional patterning after 35 days *in vitro*. Briefly, iPSCs were dissociated to single cells using Accutase™ and plated on Matrigel in STEMdiff™ SMADi Neural Induction Media +  10 μM Y-27632. Media changes subsequently occurred daily without Y-27632, and cells were passaged using Accutase™ every 7 days for 21 days during neural induction. Neural stem cells were re-plated to 15 μg/mL poly-L-ornithine and 10 μg/mL mouse laminin coated plates and patterned in STEMdiff™ Midbrain Neuron Differentiation Media +  200 ng/mL recombinant human sonic hedgehog C24II (Shh) for 14 days. Despite the manufacturer including “differentiation” in the media’s name, only midbrain progenitor patterning via Shh is occurring with the aforementioned media. After midbrain neural progenitor patterning was complete, cells were cultured in STEMdiff™ Midbrain Maturation Media for an additional 2 weeks for differentiation to neuronal and glial lineage fates. Product catalog numbers and manufacturer details are available in Supporting Information S1-S2 Table.

### Morphine treatments and defining morphine withdrawal

All treatment groups and their corresponding developmental stage in infants with POE is depicted in Fig 3A, and further described here: In order to mimic chronic POE and withdrawal on the developing human brain, midbrain neural progenitors derived using the longer protocol were cultured for 5 days in STEMdiff™ Midbrain Neuron Differentiation Media, supplemented with 200 ng/mL Shh, + /- 10 μM morphine sulfate after patterning was complete. Midbrain neural progenitors were then transitioned to STEMdiff™ Midbrain Neuron Maturation Media for 5 days + /- morphine sulfate. Thus, the 10 days of morphine exposure occurs during early neuronal and glial cell fate commitments of neural progenitors. On day 10 of morphine exposure (DIV47), cells either: 1) experienced morphine washout using 1 x phosphate buffered saline (PBS without Ca^2 +^ or Mg^2+^) three times to stimulate withdrawal with subsequent culture in STEMdiff™ Midbrain Neuron Maturation Media without morphine; or 2) continued to be treated with morphine in STEMdiff™ Midbrain Neuron Maturation Media for 10 more days. Treatment Condition #1 represents the developing fetal brain without opioid exposure. Treatment Condition #2 represents uninterrupted opioid exposure throughout the developing brain’s regional patterning and differentiation process. Treatment Condition #3 represents the developing fetal brain that experiences withdrawal after a chronic period of opioid exposure. While some cell culture studies involving animal cells have previously exposed neural stem cells to morphine for 72 hours [35,83], the duration of 10 days was chosen to mimic an increased exposure window during regional specification and neuronal/glial differentiation. The supraphysiologic concentration of morphine sulfate was chosen based on: 1) previously published morphine exposure studies using this concentration [35,83,84], and 2) protracted time in monolayer culture relative to brain organoids, which can be cultured for > 9 months at physiologic concentrations. Previously published morphine dose-response curves in human and mouse neural stem cells have strong phenotype readouts at this dose, as well [83,85]. Morphine withdrawal was quantified using cAMP immunofluorescence mean fluorescent intensity (MFI) 24 hours after washout [77]. Quantifying levels of cAMP via immunocapture is a standard method for quantifying morphine withdrawal in neural cells [86,87].

**Fig 1:**
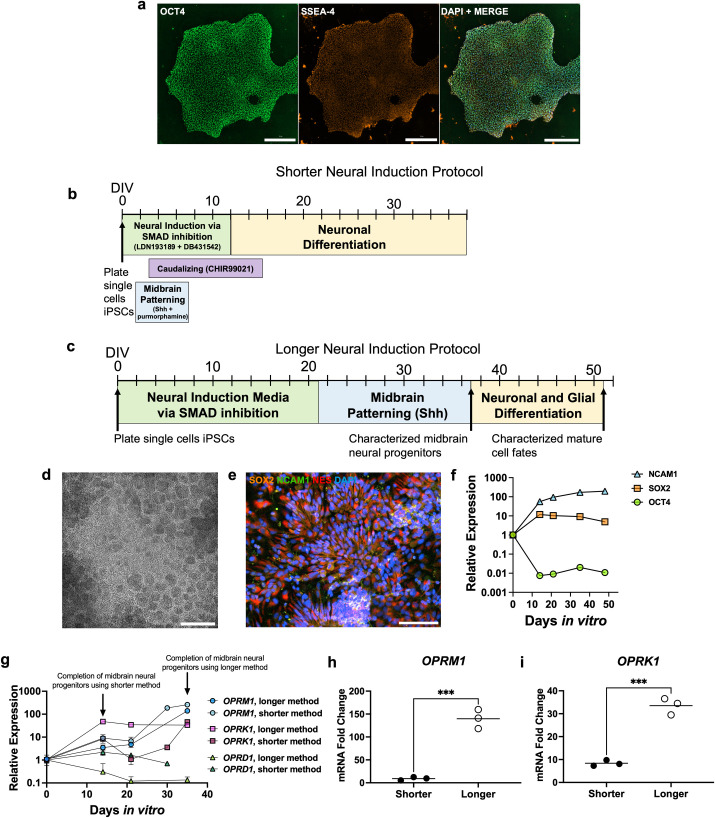
Characterization of opioid receptor expression in iPSC-derived neural stem cells using two different neural induction and patterning methods. SCTi003-A human iPSC colony expresses markers of pluripotency, OCT4 and SSEA-4. Scale bars are 300μm (a). Shorter and longer method neural induction, midbrain patterning with Shh, and differentiation paradigms, respectively (b, c). Neural rosette formation at DIV7 in brightfield. Scale bar is 300 μm (d). DIV21 immunofluorescence staining of neural stem cells derived from longer method by SOX2 (orange), NCAM1 (green), NES (red), and DAPI (blue). Scale bar is 100 μm (e). Relative expression of NCAM1, SOX2, and OCT4 over longer protocol from RT-qPCR data. Each data point represents the mean of 3 biological replicates. (f). Canonical opioid receptor relative expression by RT-qPCR over time between shorter and longer neural induction methods. Each data point represents the mean of 3 biological replicates (g). Comparison of *OPRM1* (Student two-tailed t-test, p =  0.0004) (h) and *OPRK1* (Student two-tailed t-test, p =  0.0003) (i) level increases at the same developmental midbrain progenitor timepoint between the two protocols (DIV13 vs. DIV35), relative to iPSC levels. Data represent mean of 3 independent experiments. GAPDH was used as a reference gene for all qPCR.

**Fig 2:**
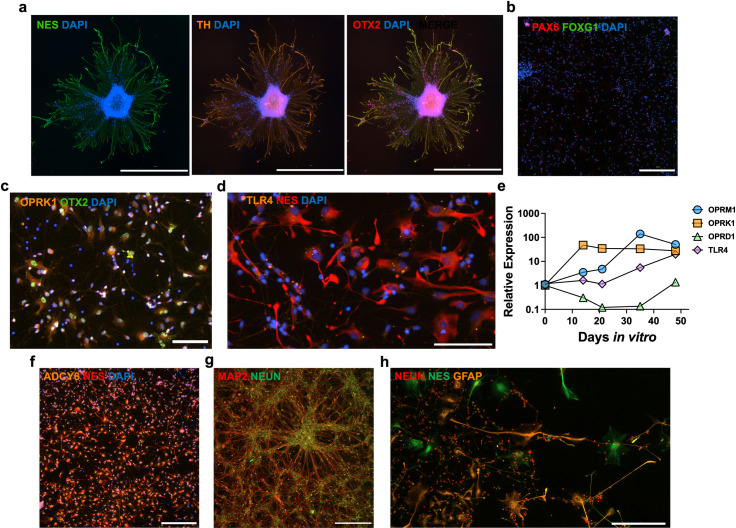
Human iPSC-derived midbrain neural progenitors express cellular, regional, and opioid signaling markers. Midbrain neural progenitors derived from the longer neural induction method can form neurosphere-like aggregates in monolayer that express NES (green), TH (orange), and OTX2 (red). Scale bar is 1000 μm (a). Progenitors lack expression of cortical progenitor markers PAX6 (red) and FOXG1 (green). Scale bar is 300 μm (b). OPRK1 (orange) is co-expressed by OTX2^ +^ progenitors. Scale bar is 100 μm (c). There is scant TLR4 expression (orange) localized around the nucleus of NES^ +^ (red) progenitors on DIV35. Scale bar is 100 μm (d). Relative expression of *OPRM1*, *OPRK1*, *OPRD1*, and *TLR4* quantified by RT-qPCR throughout the longer neural induction and patterning method. Each data point represents the mean of 3 biological replicates (e). NES^ +^ (red) progenitors express ADCY8 (orange) at DIV35. Scale is 300 μm (f). Immature neurons are MAP2^ +^ (red) and NEUN^ +^ (green) at DIV50. Scale is 300 μm (g). GFAP^ +^ astrocytic precursors (orange) develop concomitantly in co-culture with NEUN^ +^ (red) neurons and NES^ +^ (green) progenitors. Scale is 200 μm (h).

**Fig 3:**
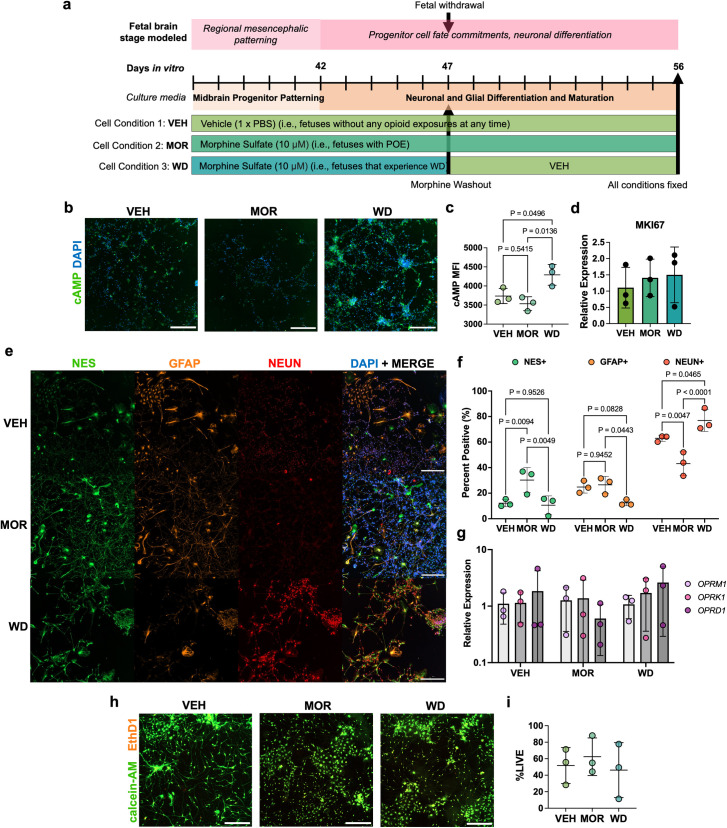
Chronic morphine exposure and withdrawal alters resulting proportions of lineage-specific midbrain progenitor cell fates. Chronic morphine exposure and withdrawal treatment paradigm of midbrain neural progenitors derived from longer neural induction and patterning method (a). Differentiating neural progenitors were fixed and immunostained 24 hours after morphine withdrawal for intracellular cAMP (n = 3). Scale bars are 300 μm (b). Biotek Cytation 5 quantified cAMP mean fluorescent intensity values across conditions. cAMP MFI was significantly higher in WD cells compared to VEH and MOR treated cells (1-way ANOVA with Tukey’s multiple comparison, F =  9.411, p =  0.014). (c). There were no differences in relative expression of marker of proliferation Ki67 between conditions (1-way ANOVA, F =  0.2614, p =  0.7783) (d). On day 56, differentiated cells were fixed and immunostained for cell identity markers NES (green), GFAP (orange), and NEUN (red). All images were taken at 10x. Scale bar is 300 μm (e). Cell identity was determined by thresholding mean staining intensities for each DAPI^ +^ cell using Biotek Cytation5. Proportions were determined by dividing the total number of NES^ + ^, GFAP^ + ^, or NEUN^ +^ cells over total number of DAPI^ +^ cells. There was a statistically significant interaction between condition and cell marker on proportions of differentiated cell fates (2-way ANOVA, F(4,18) =  16.05, p <  0.0001) (f). There were no changes to canonical opioid receptor transcript levels as a result of chronic morphine exposure and withdrawal (2-way ANOVA, F(4,18) =  0.569, p =  0.688) (g). LIVE/DEAD™ staining was performed on live, unfixed cells 24 hours after morphine withdrawal. Scale bar is 300 μm (h). There was no difference in proportions of calcein-AM^ +^ cells between treatment conditions (1-way ANOVA, F =  0.292, **p** =  0.756) (i). All error bars represent the mean + /- 1 standard deviation.

### Quantitative immunocytochemistry

Cells were fixed in chilled 4% paraformaldehyde (PFA) for 10 minutes within cell culture plate wells and washed three times in 1 x PBS. Permeabilization for intracellular epitopes was performed using 0.25% Triton X-100 detergent for 5 minutes, followed by three washes in 1 x PBS. Samples were blocked with 10% bovine serum albumin (BSA) for 30 minutes at room temperature. Primary and secondary antibodies were prepared in 1 x PBS +  0.1% Tween 20, and 1% BSA. Primary antibodies incubated on samples at 4°C overnight, and secondary antibodies incubated for 1 hour at room temperature. Antibody manufacturer, catalog numbers, and working dilutions can be found in Supporting Information. A Biotek Cytation 5 Cell Imaging Multimode Reader (Agilent, Santa Clara) was used for immunofluorescence microscopy imaging and MFI quantification. At least 5 technical replicates and 3 biological replicates were used for each treatment condition. Cellular identity was auto-calculated using intensity thresholding parameters specific to each cell type for all DAPI^ +^ cells. Resulting cell type proportions were calculated from cell thresholds using an in-house R script, “CellFateQuant”, available on GitHub at https://github.com/eraich26/CellFateQuant. All images were taken at 10X magnification unless otherwise noted.

### Reverse transcription and qPCR

At various time points, total RNA was extracted using the miRNeasy kit (Qiagen, Hilden, Germany). cDNA was generated using the SuperScript™ First-Strand Synthesis System for RT-qPCR (Invitrogen™, Waltham). PowerUp™ SYBER™ Green Master Mix (Applied Biosystems™, Waltham) was used for qPCR on a QuantStudio™ 5. All mRNA primer sequences were used from the freely accessible Harvard Primer Bank, which has experimentally optimized thousands of PCR primer sequences [88]. All sequences are listed in the Supporting Information section. Relative expression was calculated using the 2^-ΔΔCt^ method. GAPDH was used as an internal control.

### LIVE/DEAD cytotoxicity assay

To assess for differences in human midbrain neural progenitor viability in morphine exposure and withdrawal, the LIVE/DEAD™ Viability/Cytotoxicity Kit (Invitrogen™, Waltham) was used. The protocol and calculations were completed according to manufacturer instructions. Calein-AM and ethidium homodimer-1 (ETHD1) uptake imaging was done on Biotek Cytation 5.

### Statistics

Three conditions of human iPSC-derived neural progenitors were compared: neural progenitors treated with vehicle (Condition #1), morphine (Condition #2) or morphine withdrawal (Condition #3) (Fig 3a). All statistics were carried out in GraphPad Prism 10. One-way ANOVAs with Tukey’s post-hoc test were used to compare differences in MFI in cAMP immunofluorescence imaging, cell viability, and *MKI67* gene expression analyses. Two-way ANOVAs with Tukey’s correction were used analyze the interaction of 1) cell fate identity with treatment condition and 2) treatment condition with levels of canonical opioid receptor transcripts.

## Results

### Longer neural induction and midbrain regional patterning steps increases neural stem cell expression of OPRM1 and OPRK1 compared to shorter protocol

Human iPSCs colonies (SCTi003-A) expressed pluripotency protein markers OCT4 and SSEA-4 (Fig 1a). Protocol schematics for both short and long neural induction and patterning protocols are shown in Fig 1b and Fig 1c, respectively. Neural induction using the longer method resulted in neural rosette formation by 7 days *in vitro* (DIV) (Fig 1c, 1d). Neural stem cells at DIV14 through DIV21 exhibited high expression of SRY-box 2 (SOX2), which slowly decreased with midbrain patterning and neuronal differentiation (Figs 1e, 1f). Neural cell adhesion marker 1 (NCAM1) was also present as early as DIV14, and steadily increased in expression during midbrain progenitor patterning and neuronal differentiation (Figs 1e, 1f). *OCT4* mRNA decreased by 100-fold by DIV14 using the longer neural induction method (Fig 1f). The shorter neural induction and patterning protocol characterized the presence of appropriate cellular and regional markers in neural progenitors in-depth previously [82]. The two monolayer methods were compared for their neural stem cell and midbrain neural progenitor gene expression of canonical opioid receptors, *OPRM1*, *OPRK1*, and *OPRD1* (Fig 1g). The shorter method optimized the time to dopaminergic neuron generation. Thus, midbrain neural progenitors generated by that protocol have completed neural induction and regional patterning by DIV13 [82], while neural progenitors derived from the longer method reach the same developmental timepoint by DIV35 (Fig 1c). Comparing relative expression at the same developmental midbrain progenitor stage (DIV13 vs. DIV35), the longer method increased *OPRM1* levels on average by about 140-fold compared to an average 9-fold increase by the shorter method (Student two-tailed t-test, *p* =  0.0004) (Fig 1h). Similarly, *OPRK1* levels increased on average by 33-fold using the longer method, whereas levels only increased on average by 8-fold by the shorter method (Student two-tailed t-test, *p* =  0.0003) (Fig 1i). *OPRD1* levels increased in midbrain neural progenitors derived from the shorter method by 2-fold compared to levels that decreased in midbrain progenitors derived by the longer method (Student two-tailed t-test, *p* =  0.0114) (S1 Fig). No *OPRD1* was detected in the shorter method beyond DIV30.

### Midbrain neural progenitor cells derived from longer neural induction method express relevant cellular, regional, and opioid signaling markers

The longer neural induction and regional patterning method generated iPSC-derived midbrain neural progenitors with higher levels of *OPRM1* and *OPRK1* than those derived from the shorter method (Figs 1g–1i), thus all subsequent characterizations and opioid exposure experiments were performed on midbrain progenitors derived from the longer method. These progenitors can form neurosphere-like aggregates in monolayer, with neurites projecting outwards (Fig 2a). Immunofluorescent microscopy revealed consistent expression of progenitor identity marker nestin (NES), plus midbrain regional markers tyrosine hydroxylase (TH), orthodenticle homeobox 2 (OTX2) (Fig 2a), and LIM homeobox transcription factor 1α (LMX1A) (S2a Fig). These midbrain progenitors have an absence of cortical progenitor markers paired box 6 (PAX6) and forkhead box G1 (FOXG1) (Fig 2b, S2b Fig). Absence of PAX6 mRNA at the midbrain progenitor stage was also confirmed by RT-PCR (S2c Fig). OTX2^ +^ neural progenitors co-express OPRK1 (Fig 2c). There is scant TLR4 protein expression localized primarily around the nucleus on NES^ +^ cells (Fig 2d). At the midbrain progenitor stage (DIV35), *OPRD1* has the lowest transcript levels out of the canonical opioid receptors, followed by *TLR4* (Fig 2e). By the end of neuronal and glial maturation (DIV48), *OPRM1, OPRK1*, *TLR4, and OPRD1* had fold change increases of 50.8, 27.6, 19.7, and 1.3, respectively (Fig 2e). In addition to surface and cytosolic opioid receptors, NES^ +^ midbrain neural progenitors also express factors that play a critical role in morphine withdrawal, such as adenylyl cyclase 8 (ADCY8) (Fig 2f). After differentiation is complete (DIV50), cells of neuronal lineage are microtubule-associated protein 2 (MAP2)^ +^ and NEUN^ +^ (Fig 2g). Additionally, there are GFAP^ +^ cells, a marker of astrocytic lineage, present in co-culture with neurons and residual NES^ +^ progenitors (Fig 2h). There was an absence of GABAergic marker, glutamate decarboxylase 2 (GAD2) on neurons (S2a Fig). Cells of neuronal lineage are neural cell adhesion molecule (NCAM) positive, which indicate neuronal immaturity [89]. Cells of astrocytic lineage also are glutamate aspartate transporter (GLAST) positive (S2d Fig) [90]. There was no amplification of oligodendrocyte transcription factor 1 (*OLIGO1)* mRNA by DIV50 (S2c Fig) nor PDGRFα^+^ oligodendrocytes (S2e Fig).

### Resulting proportions of differentiated neurons, astrocytes, and progenitors are altered in chronic morphine exposure and withdrawal

After completion of midbrain neural progenitor patterning with Shh on DIV37 (Fig 1b), progenitors were split into three treatment conditions, as shown in Fig 3a. Neural progenitors that experienced morphine withdrawal had higher average mean fluorescent intensities (MFI) of cAMP than those treated with only vehicle (*p* =  0.0496) or morphine (*p* =  0.014) (one-way ANOVA with Tukey’s multiple comparison, *F* =  9.411, *p* =  0.014) (Fig 3b, 3c). Chronic morphine exposure and withdrawal did not significantly affect transcript levels of marker of proliferation Ki67 (*MKI67)* 24 hours after morphine withdrawal (one-way ANOVA, *F* =  0.2614, *p* =  0.7783) (Fig 3d). On day 56, differentiated cells were fixed and immunostained for cell fate identity markers of immature neurons (NEUN), astrocytic precursors (GFAP), and progenitors (NES) (Fig 3e). The impact of treatment condition on resulting cell fates (% of cells positive for identity marker) was assessed. There was a significant interaction between treatment condition and cell fate on proportions of marker positive cells (two-way ANOVA, *F*(4,18) =  16.05, *p* <  0.0001) (Fig 3f). Chronic morphine exposure significantly increased proportions of NES^ +^ cells compared to VEH by 18% (mean proportion of 12.2% vs. (30.2%) (Tukey’s multiple comparisons, adj. *p* =  0.009), whereas morphine withdrawal allowed proportions to return to VEH levels (mean proportion of 10.6% vs. 12.2%) (adj. *p* =  0.9526). Chronic morphine exposure did not alter proportions of resulting GFAP^ +^ cells compared to VEH (mean proportion of 24.8% vs. 26.5%) (adj. *p* =  0.9452), although morphine withdrawal decreased proportions of GFAP^ +^ cells relative to morphine exposed cells by 14% (mean proportion of 26.5% vs. 12.5%). There was not a significant difference in GFAP^ +^ cells in the condition that experienced morphine withdrawal compared to VEH (mean proportion of 24.8% vs. 12.5%) (adj. *p* =  0.083). Chronic morphine exposure significantly decreased proportions of NEUN^ +^ cells compared to VEH by 19.81% (mean proportion of 62.9% vs. 43.16%) (adj. *p* =  0.005), whereas morphine withdrawal significantly increased the proportion of NEUN^ +^ cells compared to VEH by 13.9% (mean proportion of 76.9% vs. 62.97%) (adj. *p* =  0.047) and MOR by 33.8% (76.9% vs. 43.16%) (*p* <  0.0001). Transcript expression of canonical opioid receptors remained statistically unchanged between treatment conditions (two-way ANOVA, *F*(4,18) =  0.569, *p* =  0.688) (Fig 3g). Differences in resulting cell fate proportions were not due to differences in cell viability induced by morphine exposure or withdrawal (one-way ANOVA, *F* =  0.292, *p* =  0.756) (Figs 3h, 3i).

## Discussion

Our current study demonstrates that choice of human iPSC neural induction and regional patterning lengths in monolayer may be important to consider when studying effects of chronic opioid exposure and withdrawal. Longer vs. shorter neural induction and regional patterning periods may result in drastic differences of canonical opioid receptor expression at the neural progenitor stage. We showed that a protocol with a longer neural induction and patterning step increased *OPRM1* and *OPRK1* levels at the same developmental time point (DIV13 in shorter method vs. DIV35 in longer method) by 130-fold and 25-fold, respectively, compared to a shorter protocol. Conversely, if opioid exposure studies are focused on the role of OPRD1 in neural stem development, the shorter neural induction and patterning method may be a better protocol choice. The observed decrease in *OPRD1* expression for both long and short methods of neural induction is in line with its low developmental expression. Multiple studies have shown that *OPRD1* expression is low to absent (relative to *OPRM1* and *OPRK1*) early in brain development and only increases in expression after birth [69,91,92].This observation further supports our model capturing brain development within the prenatal period. Further, there is ample evidence that OPRD1 is involved in higher processes of learning, memory, and emotions; thus, OPRD1 expression may increase postnatally to regulate these developmental processes [93]. Interestingly, *OPRD1* expression increased over an *in vitro* cultring period of 100 days in 3D hiPSC-derived mibrain organoids [71]. It’s possible that OPRD1 expression is dependent on the 3D cell-cell interactions that occur in an organoid. Given the importance of opioid receptor expression in POE pathophysiology [94,95], differentiation protocols should be chosen for optimal receptor expression.

To our knowledge, this is the first study to show TLR4 expression in human iPSC-derived midbrain neural progenitors, and how it changes during regional patterning and neuronal maturation. Establishing the presence of TLR4 in human stem cell models of opioid exposure is paramount to understanding early developmental effects of opioid-induced neuroinflammation. TLR4 is specifically relevant to POE and NOWS due to its ability to bind to morphine and other opioids [31,96]. We showed that TLR4 has lower expression compared to OPRM1 and OPRK1, which recapitulates expression profiles observed in the fetal midbrain [97]. Given the scant immunofluorescence staining of TLR4, further work is needed to identify if TLR4 is localized to the membrane or cytosolic fractions of iPSC-derived midbrain neural progenitors. Further, we showed that *OPRK1* has highest expression earliest in neural stem cells, followed by *OPRM1*. These results parallel similar trends in the embryonic mouse brain, with *OPRM1* and *OPRK1* transcripts being first detectable in the mouse midbrain and basal ganglia [92]. Thus, this human model system may be used to untangle receptor-specific effects at different developmental time points in the midbrain.

Finally, we showed that morphine exposure and subsequent withdrawal alters proportions of resulting cell fates. These effects were not due to differences in chronic morphine-induced cytotoxicity or proliferation, which is in line with findings of previous studies [98,99]. Chronic morphine exposure increased the proportions of NES^ +^ progenitors at the expense of mature NEUN^ +^ cells of neuronal lineage. Morphine withdrawal allowed proportions of progenitors to return to those of the VEH treated group. This result recapitulated trends found by Kim *et al* [71]. Interestingly, morphine withdrawal also increased the proportion of mature NEUN^ +^ cells, relative to both VEH and MOR treated groups at the expense of GFAP^ +^ cells. While previous studies have consistently shown that morphine stalls maturation of early neuronal progenitors [71,100,101], there has been less data on how morphine withdrawal affects neurodevelopment. One study showed that administration of naloxone for 10 days on rat neural progenitors after OPRM1 and OPRD1 agonist incubation induced a 3-fold increase in neuron-preferential differentiation, while reducing differentiation into astrocytes by 3-fold and oligodendrocytes by 50% [102]. In our study, one-time morphine washout to stimulate withdrawal was sufficient to replicate these trends. Another published study showed that 4 days of morphine exposure on mouse neural progenitors preferentially differentiated into GFAP^ +^ cells rather than cells of neuronal lineage, and that this process was regulated by microRNA-181a [103]. In our study, chronic morphine exposure had no effect on proportions of GFAP^ +^ cells, though this could be due differences in treatment length or use of an animal experimental model. It remains unclear how these neurodevelopmental alterations may manifest in infants with POE and NOWS, though observed reduced head circumferences [5] and decreased myelin basic protein [104] could be related to the lineage-specific cell fate changes observed here. Neurogenesis and brain cell fate decisions continue from the prenatal to neonatal phase [105,106]; thus, prolonged NOWS pharmacotherapy treatment with morphine may impact proportions of cell fates in the developing brain. Taken together, the effects of chronic opioid exposure on developing neural progenitors are not benign. Further mechanistic research and analyses with greater cellular resolution is warranted to understand how human lineage-specific changes in progenitor differentiation are altered by opioid exposure.

There are several limitations of this study. The length of differentiation in the longer protocol is a factor to consider when evaluating availability of resources and time. Despite being able to co-culture NEUN^ +^ neurons, GFAP/GLAST^ +^ cells, and NES^ +^ progenitors together, other cell types that could be supported by a 3D culture system (i.e., oligodendrocytes, microglia, pericytes) were absent [71]. Additionally, our iPSC line was a female healthy control line, derived from an adult with calculated 78.2% European and 21.8% South Asian ancestry. While there have been calls for including more female and ethnically diverse iPSC lines in models of POE [53], we did not include a male line for comparison of sex-specific effects. Further, we used an adult-derived hiPSC line to model a disorder in fetuses, which could have affected the epigenetic age of the line. Although, it has been shown that somatic reprogramming resets most of the epigenome and increased passaging of iPSCs ( > 20 passages) dimishes retained age-related epigenetic signatures [107]. Additionally, it has been shown that hiPSC-derived neural progenitors and neurons from adult/adolescent donors have an epigenetic signature which correlates with that of the 1^st^ trimester fetal brain [108]. Future studies may want to profile the DNA methylation signatures of iPSC lines, or use an iPSC line from a neonatal donor. We used only 1 cell line in this work, so our results serve as a proof of concept for future studies. Future studies may also want to include use of a larger number of cell lines to further advance our understanding of the effects opioid exposure and withdrawal have on neurogenesis during early development.

To our knowledge, this is the first 2D human iPSC-derived midbrain neural stem cell model of POE and withdrawal. The strength of using 2D human iPSC neuronal stem cell models is that opioid exposures can be modeled at critical, regional specific, developmental checkpoints with precision and ease of genomic manipulation. Future studies could apply this model to understand how molecular mechanisms determine cell fate in response to morphine exposure and withdrawal.

## Supporting information

S1 Table Cell culture and immunocytochemistry product information. (ZIP)

S1 Fig Shorter neural induction and patterning protocol increases OPRD1 levels in midbrain neural progenitors more than the longer protocol. At completion of midbrain neural progenitor patterning for each protocol, *OPRD1* levels decreased in the longer protocol (DIV35) relative to iPSC levels (mean fold change = 0.336), compared to an increase (mean fold change of 2.19) in the shorter protocol (DIV13) (Student t-test, two-tailed, p = 0.0114).
(TIF)

S2 Fig Further characterizations of iPSC-derived midbrain neural cell types using the longer neural induction and regional patterning protocol. In co-culture with MAP2+/GAD2^-^ immature neurons, neural progenitors are LMX1a^+^ (DIV50) (a) Green punctate artifacts do not overlap with cells. There is an absence of PAX6 staining of TH^+^ midbrain progenitors (b). No PAX6 mRNA amplified in midbrain neural progenitors (DIV35) and no OLIGO1 mRNA amplified on DIV50 using experimentally validated primers in RT-qPCR (left panel). Associated melt curves are shown in the panel on the right. (c). There is presence of GLAST^+^ (orange) astrocytic precursors, and NCAM^+^ immature neurons (DIV50) (red). Scale bar = 200 μm (d). At completion of differentiation of human midbrain neural progenitors (DIV50), there is an absence of PDGRFα (orange) within co-culture of OTX2^+^ (green) and NES^+^ (red) neural progenitors. Scale bar = 300 μm (e).
(TIF)

S2 Table Reported Figure Data. (ZIP)
