# Supplementary figures and images for: A human iPSC-derived midbrain neural stem cell model of prenatal opioid exposure and withdrawal: A proof of concept study
Source: PLoS One. 2025 Apr 1;20(4):e0319418. doi: 10.1371/journal.pone.0319418 (PMC11960892; doi:10.1371/journal.pone.0319418)

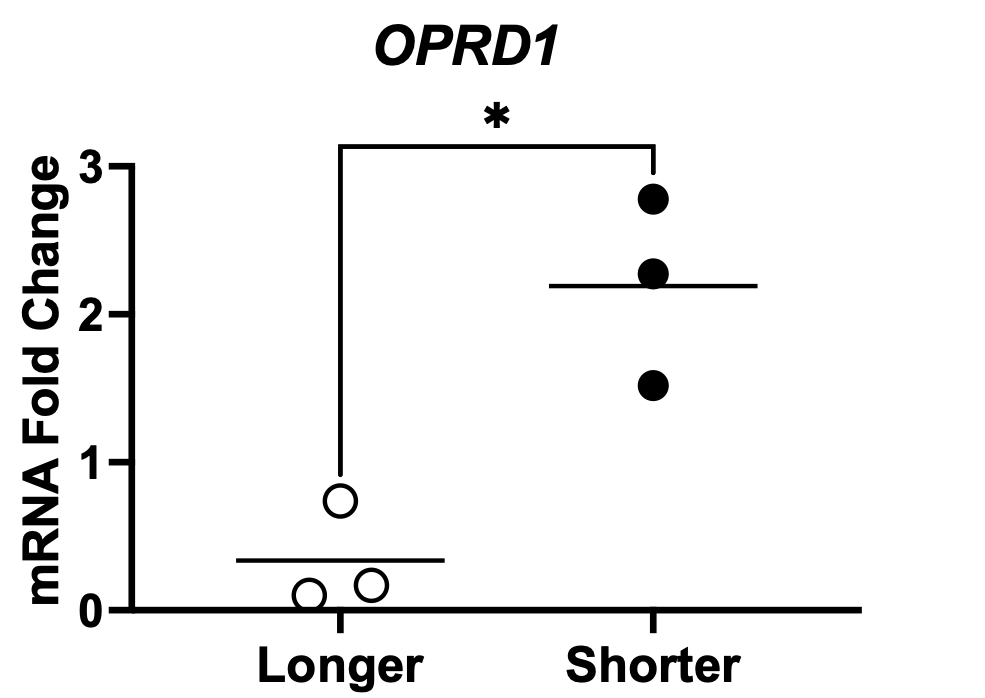

Supplement: S1 Fig — At completion of midbrain neural progenitor patterning for each protocol, OPRD1 levels decreased in the longer protocol (DIV35) relative to iPSC levels (mean fold change = 0.336), compared to an increase (mean fold change of 2.19) in the shorter protocol (DIV13) (Student t-test, two-tailed, p = 0.0114). (TIF) [file pone.0319418.s002.tif]

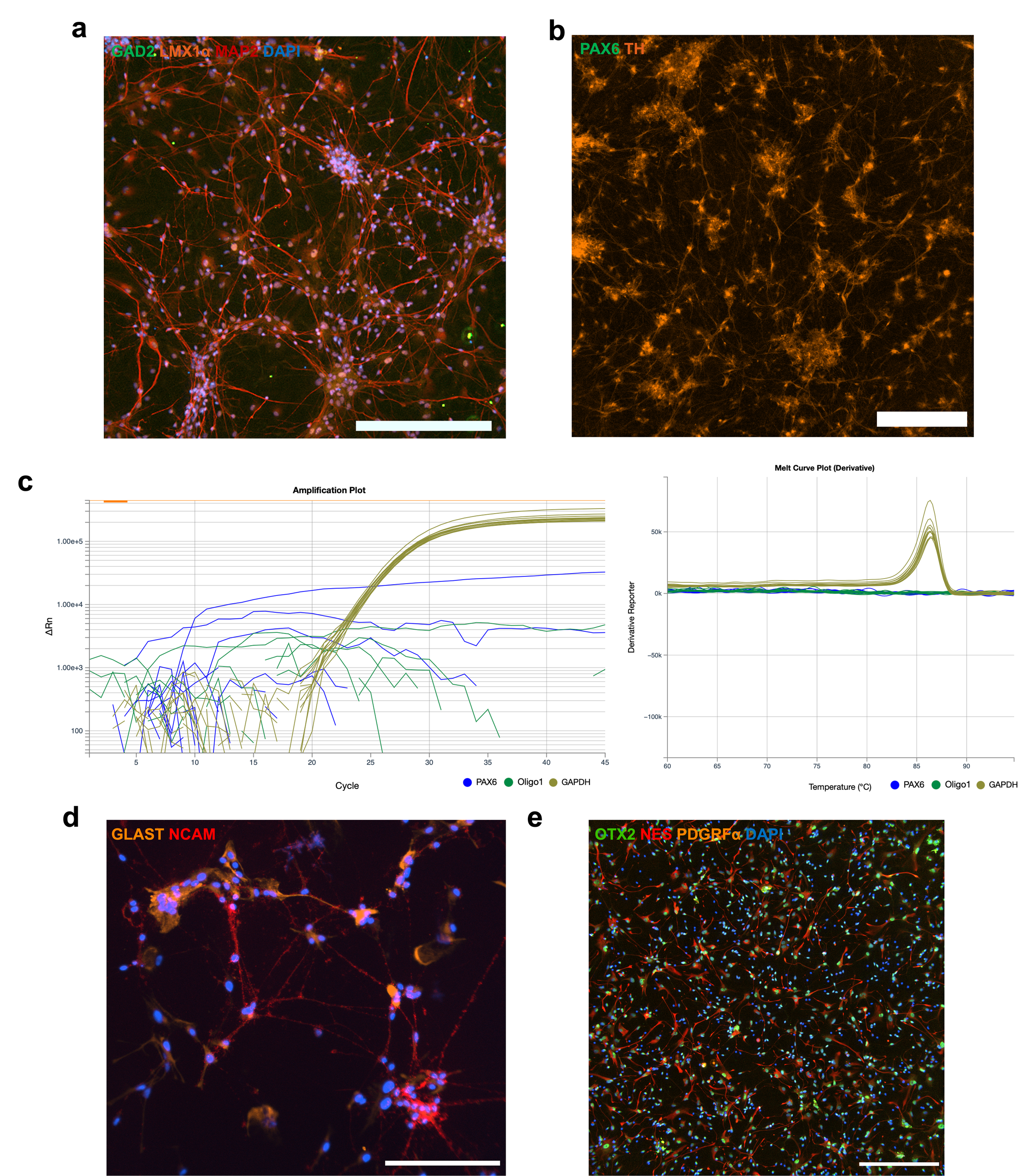

Supplement: S2 Fig — In co-culture with MAP2+/GAD2- immature neurons, neural progenitors are LMX1a+ (DIV50) (a) Green punctate artifacts do not overlap with cells. There is an absence of PAX6 staining of TH+ midbrain progenitors (b). No PAX6 mRNA amplified in midbrain neural progenitors (DIV35) and no OLIGO1 mRNA amplified on DIV50 using experimentally validated primers in RT-qPCR (left panel). Associated melt curves are shown in the panel on the right. (c). There is presence of GLAST+ (orange) astrocytic precursors, and NCAM+ immature neurons (DIV50) (red). Scale bar = 200 μm (d). At completion of differentiation of human midbrain neural progenitors (DIV50), there is an absence of PDGRFα (orange) within co-culture of OTX2+ (green) and NES+ (red) neural progenitors. Scale bar = 300 μm (e). (TIF) [file pone.0319418.s003.tif]
